# Fatal injuries and economic development in the population sample of Central and Eastern European Countries: the perspective of adolescents

**DOI:** 10.1007/s00038-020-01449-5

**Published:** 2020-08-06

**Authors:** Michal Miovsky, Beata Gavurova, Viera Ivankova, Martin Rigelsky, Jaroslav Sejvl

**Affiliations:** 1 Department of Addictology, First Faculty of Medicine, Charles University in Prague, Prague, Czech Republic; 2 General University Hospital in Prague, Prague, Czech Republic; 3 Faculty of Management, University of Prešov in Prešov, Prešov, Slovakia; 4 Center for Applied Economic Research, Faculty of Management and Economics, Tomas Bata University in Zlin, Mostni 5139 760 01 Zlin, Czech Republic

**Keywords:** Injury mortality, Economic growth, Human development, Gender differences, Age-specific differences, Epidemiologic transition

## Abstract

**Objectives:**

Researches consider the young generation (adolescents) to be the population group whose mortality from injury has the lowest effect on economic growth. The objective was to evaluate the relations between economic indicators and preventable injury mortality in Central and Eastern European Countries (CEECs), with a primary focus on adolescents.

**Methods:**

The analyses included health indicators of preventable injury mortality and economic indicators that represent human development and economic growth in the CEECs from 1990 to 2016. The analytical process involved a population group divided by age (0–14 years: children, 15–24 years: adolescents, 25–74 years: adults) and gender. Descriptive analysis, cluster analysis and primarily panel regression analysis were used.

**Results:**

Significant effects of economic indicators on drowning were found in all analysed relations. In the group of adolescents, significant effects of fatal falls were found. Overall, it can be concluded that the effects of fatal injuries are not homogenous between age and gender groups.

**Conclusions:**

The effects of years and individual countries should be taken into account in the cross-sectional analyses. In terms of economic growth, public policies should focus on drowning in children, on falls in adolescents and on transport accidents, fire injuries and poisoning in adults.

## Introduction

Injuries are considered as one of the main causes of the global burden of disease (Krug et al. 2000; Haagsma et al. 2016), it is also known that injuries affect mostly younger population and often cause long-term disability or death (Bachani et al. 2017; Mehmood et al. 2017). Although the number of deaths due to injuries has decreased (Molcho et al. 2015; Pakkari et al. 2016), this issue can be considered very important from an economic point of view. Injury mortality has been widely studied in different countries (Mack et al. 2017; Moniruzzaman 2018), and deaths from intentional and unintentional injuries are also evident in European countries (Stone et al. 2006; Petridou et al. 2007). Evidence shows an increasing trend in mortality due to falls, poisoning, suffocation and intentional injuries (Paulozzi et al. 2006; Hong et al. 2011; Pham et al. 2018), and a decreasing trend in mortality due to drowning, burning and transport-related injuries (Hong et al. 2011; Melchor et al. 2015; Sadeghian et al. 2019; Nguyen et al. 2020).

The above-mentioned findings arouse interest in a more detailed knowledge of the differences among age and gender groups in this issue. Cunningham et al. (2018) revealed apparent inequalities in child and adolescent mortality due to injuries such as transport accident injuries, fire or burn injuries, drowning, suffocation. The findings of this study confirmed gender differences in all of mentioned causes of deaths, mainly in drowning and suffocation deaths. Female children and adolescents showed more positive outcomes and these gender differences were lower among children than among adolescents. This is in line with the fact that adolescents are more likely to take risks compared to children or adults (Steinberg 2004, 2007). Simultaneously, it should be emphasized that self-harm and suicide are major problems in adolescents (Hawton et al. 2012). Ray et al. (2020) examined mortality due to injuries among children aged 1–14 years and adolescents aged 15–24 years, their results also confirmed gender inequality with advantage for females. The authors highlighted that accidental poisoning and transport accidents were the dominant causes of death of males and females in both age groups, and emphasized that injury mortality pronouncedly increased after age 15 years, especially among males. At the same time, the findings provided by Corso et al. (2006) revealed that males showed a greater number of fatal injuries than females in all age categories (0–74 years), especially in the group of adolescents (aged 15–24 years). With a focus on a number of specific fatal injuries, males showed more negative results in falls, fire and burn, poisoning and, in particular, drowning. In general, a higher rate of injury mortality of males was confirmed in several other studies (Sorenson 2011; Yin et al. 2015; Dandona et al. 2020).

Based on the above-mentioned findings, the injury mortality is a global health problem that can affect the economic condition of countries. Fatal and non-fatal injuries represent a huge health and financial burden that can be reflected in higher health cost and lost productivity (Corso et al. 2006), while a higher severity of injuries means higher costs (Geraerds et al. 2019). Harlan et al. (1990) revealed that young adults (aged 17–44 years) caused the highest health cost of injury, while males indicate a greater proportion of the total cost than females. In terms of health costs and productivity losses, very similar findings were later confirmed by Corso et al. (2006), who highlighted that productivity losses represent 80% of the total injury cost. The authors also revealed that males showed the dominant part of total productivity losses (73%), mainly due to their higher injury mortality rate.

It should be noted that the relations between injury mortality and economic condition have been examined only in older studies and the following findings have been demonstrated. Plitponkarnpim et al. (1999) confirmed an inverse relation between injury mortality of children aged 1–14 years and Gross National Product (GNP) per capita, on the scale of the global level. Ahmed and Andersson (2000) revealed an inverse relation between fatal injuries and GNP per capita, while stronger relationship was identified in a group of adults aged 35–44 years compared to the group of adolescents aged 15–24 years. Moniruzzaman and Andersson (2005) investigated this relation among older adults and seniors, their results showed that a decline in injury mortality of females and males aged 45–74 years causes an increase in GNP per capita, and in the case of the population over 75 years, there is a moderate positive relation. These results indicate substantial findings for economic theories, but these data need to be updated and verified in the current conditions.

From the opposite view of this issue, Trujillo et al. (2010) confirmed the relations between economic growth represented by gross domestic product (GDP) and fatal injuries among seniors, and their findings showed a moderate effect. Based on a negative association, a growth in GDP can reduce fatal injuries due to traffic accidents, suicides and homicides, and a positive association was identified between economic growth and fatal injuries due to falls. The authors also emphasized that a stronger negative association between economic growth and fatal injuries can be found among the younger generation. Muazzam and Nasrullah (2011) considered the GDP as a significant factor of injury mortality and revealed that most male injury mortality decreased with increasing GDP, except motor-vehicle traffic crashes. Similar results were found in females, except suicides. These findings are in line with the general fact that improvements in economic conditions reduce mortality in developed and less developed countries (Birchenall 2007, O’Hare et al. 2013). Simultaneously, Zakaria et al. (2020) revealed that human capital, income per capita and urbanization are factors that reduce child mortality.

Despite these economic dimensions, it remains unanswered whether there is a relation between human development index (HDI) and fatal injuries. From an economic point of view, the injury mortality can be seen as a loss of potential economic productivity, performance, as well as prosperity in countries. Adolescent mortality is a health burden (Kassebaum et al. 2017) and can also be an economic burden, as this part of the population, in which resources have already been invested, represents a group of people of working age in the near future. At present, it is possible to discuss the effect of human development on the preventable injury mortality of adolescents and, consequently, how this mortality affects the economic growth.

## Methods

The objective of the study is to evaluate the relations between economic indicators and preventable injury mortality in the CEECs in age and gender specifications, with a primary focus on adolescents. This study deals with the issue of the effect of human development on the frequency of fatal injuries and, consequently, the effect of these deaths on economic growth represented by GDP. These effects were assessed among age and gender groups.

The aim was achieved through the analyses in a sample of CEECs, i.e. Albania, Bulgaria, Croatia, Czechia, Hungary, Poland, Romania, Slovakia, Slovenia, Estonia, Latvia, Lithuania. Two categories of data were used in the analyses: (1) economic variables: Human Development Index (HDI) obtained from the databases of United Nations Development Programme (UNDP 2020) and Gross Domestic Product (GDP) per capita in current USD obtained from the databases of The World Bank (WB, 2020); (2) health variables of preventable injury mortality (PCMi): transport accidents (TRANSPORT), falls (FALLS), accidental drowning and submersion (DROWNING), exposure to smoke, fire and flames (FIRE), accidental poisoning by and exposure to noxious substances (POISONING), intentional self-harm (SELF-HARM), which were obtained from the database of World Health Organization (WHO 2020). Injury mortality indicators were standardized per 100,000 population in each group. The individual groups were divided by gender: men (MALE), women (FEMALE), the total population regardless of gender (ALL); and by age: 0–14 years (children), 15–24 years (adolescents), 25–74 years (adults). The selected health indicators belong to the category of preventable deaths. The data were collected for the years 1990–2016—available years in all variables (intersection of databases).

The following section was divided into two parts. Descriptive analysis and cluster analysis were used in the first part and regression analysis was applied in the second part. The descriptive analysis included 95% confidence interval for mean (CI) and standard deviation (SD), which identified the basic characteristics that provided a clearer picture of the analysed variables. In addition to this analysis, a difference analysis was also applied, in which the significance of the difference between years and between countries was tested using the Kruskall–Wallis rank sum test (K–W). The output was a prerequisite for the application of cluster analysis that was applied using the Ward’s method selected on the basis of the agglomerative coefficient, and the optimal number of clusters was estimated using the Silhouette method. Based on the output of the cluster analysis, a new variable was created, which was used as a panel variable in the subsequent application of regression analysis. The effects of this variable were verified using the F test for individual effects. In the case of the confirmed effect, a choice was made between a fixed effect model and a random effect model (Swamy–Arora’s transformation). The choice was supported by the Hausman Test. In the last step, the presence of significant heteroscedasticity was verified using the Breusch–Pagan test.

## Results

The first part of analytical processing consists of determining the basic statistical characteristics (CI, SD) and difference tests of economic indicators (HDI, GDP) and selected health indicators of preventable injury mortality (PCMi), between years and between countries. This information creates a more comprehensive view of the inputs and justifies the applied analyses.

The HDI took a value of 95% CI in the range of 0.77–0.78 and a value of SD was 0.06. When applying the difference test between years, the nonparametric Kruskal–Wallis test acquired a value of *χ*^2^ equals to 178.11 (< 2.2 × 10^−16^); therefore, there was a significant difference in HDI between years. In the case of testing the difference between countries, the test acquired a value of *χ*^2^ equals to 114.74 (< 2.2 × 10^−16^); therefore, a significant difference in HDI between countries was confirmed. The GDP took a value of 95% CI in the range of 7907.23–9345.023 and a value of SD was 6165.8. When applying the difference test between years, the nonparametric Kruskal–Wallis test acquired a value of *χ*^2^ equals to 183.32 (< 2.2 × 10^−16^); therefore, there was a significant difference in GDP between years. In the case of testing the difference between countries, the test acquired a value of *χ*^2^ equals to 106.08 (< 2.2 × 10^−16^); therefore, a significant difference in GDP between countries was confirmed.

Table 1 provides several information: (1) the values of central tendency (CI) that allow to compare the frequency of selected fatal injuries among age and gender groups; (2) the variability (SD) that identifies how the values differ from the mean, and this characteristics has the greatest importance in comparing the frequency of selected fatal injuries among age and gender groups; (3) the tests of the difference in the frequency of selected fatal injuries between years and between countries. In general, it can be confirmed that males had higher frequency of deaths than females in all fatal injuries. Focusing on the youngest group, i.e. children (0–14 years), there were no notable gender differences, while the greatest difference was found in transport accidents (CI-Males: 4.13–4.74; CI-Females: 2.67–3.06), the smallest difference was found in poisoning (CI-Males: 0.54–0.74; CI-Females: 0.49–0.67). In the group of adolescents (15–24 years), the gender differences were more noticeable, while the most pronounced difference was found in self-harm (CI-Males: 17.44–20.02; CI-Females: 3.48–3.95) and in drowning (CI-Males: 5.11–6.21; CI-Females: 0.70–0.87). In the group of adults (25–74 years), obvious gender differences occurred in almost all indicators, and the greatest difference was found in drowning (CI-Males: 7.24–9.03; CI-Females: 1.37–1.67). Differences were also found between age groups, while the group of children showed notably lower values in transport accidents (CI 3.43–3.91). In the case of fatal fire injuries, the group of adults had notably more deaths (CI 2.47–3.39) compared to the other two age groups, and in other cases, it can be concluded that the frequency of fatal injuries increases with age. In terms of variability, it can be noted that females showed a lower rate, i.e. the results of females are more accurate and the data of males were more scattered. The difference tests of the analysed indicators between countries showed a significant difference at the level of *α* < 0.001 in all cases except one, in which was shown a significant difference at the level of significance *α* < 0.01 (Females, 0–14 years, self-harm). Differences between individual years were found in most cases. Based on the verification of the mentioned differences, the cluster analysis was applied.

**Table 1:** Descriptive characteristics of preventable injury mortality in the countries of Central and Eastern Europe (1990–2016)

	TRANSPORT	FALLS	DROWNING	FIRE	POISONING	SELF_HARM
*0–14 M*						
CI	4.13–4.74	0.61–0.78	3.18–3.98	0.60–0.85	0.54–0.74	0.72–0.87
SD	2.62	0.72	3.43	1.08	0.93	0.67
K–Wy	152.12^†^	61.75^†^	68.53^†^	40.68*	52.04**	61.46^†^
K–Wc	54.90^†^	92.83^†^	151.79^†^	127.27^†^	108.21^†^	48.08^†^
*0–14 F*						
CI	2.67–3.06	0.35–0.46	1.23–1.56	0.48–0.70	0.49–0.67	0.24–0.32
SD	1.69	0.48	1.43	0.93	0.80	0.33
K–Wy	122.27^†^	67.68^†^	59.45^†^	43.97*	53.57**	36.81•
K–Wc	50.38^†^	54.76^†^	131.32^†^	121.52^†^	99.32^†^	30.61**
*0–14 ALL*						
CI	3.43–3.91	0.49–0.62	2.24–2.79	0.54–0.77	0.52–0.71	0.50–0.59
SD	2.05	0.54	2.34	0.96	0.82	0.41
K–Wy	153.58^†^	77.88^†^	66.91^†^	40.74*	57.89^†^	57.47^†^
K–Wc	54.52^†^	91.43^†^	161.30^†^	154.76^†^	106.17^†^	60.52^†^
*15–24 M*						
CI	27.11–30.35	2.17–2.55	5.11–6.21	0.55–0.74	3.73–5.22	17.44–20.02
SD	13.88	1.62	4.71	0.81	6.38	11.03
K–Wy	94.42^†^	48.93**	35.47	52.96**	47.65**	13.50
K–Wc	92.65^†^	97.04^†^	165.43^†^	61.79^†^	136.12^†^	227.56^†^
*15–24 F*						
CI	7.26–8.12	0.51–0.64	0.70–0.87	0.15–0.23	0.98–1.25	3.48–3.95
SD	3.97	0.55	0.76	0.36	1.14	2.03
K–Wy	99.42^†^	36.61•	58.40^†^	29.27	35.40•	55.18^†^
K–Wc	73.16^†^	50.43^†^	74.21^†^	63.34^†^	103.58^†^	108.58^†^
*15–24 ALL*						
CI	17.41–19.45	1.37–1.60	2.96–3.57	0.36–0.48	2.40–3.26	10.65–12.12
SD	8.74	0.98	2.64	0.52	3.69	6.28
K–Wy	99.89^†^	47.61**	39.02*	45.24*	49.33**	17.09
K–Wc	92.05^†^	103.50^†^	164.75^†^	75.89^†^	141.51^†^	217.20^†^
*25–74 M*						
CI	26.65–29.53	14.57–16.45	7.24–9.03	3.98–5.55	12.51–16.54	40.04–45.71
SD	12.34	8.08	7.66	6.74	17.23	24.28
K–Wy	150.62^†^	15.60	16.40	9.33	5.85	16.59
K–Wc	67.59^†^	211.67^†^	227.00^†^	249.67^†^	235.37^†^	237.88^†^
*25–74 F*						
CI	6.38–7.05	3.75–4.34	1.37–1.67	1.14–1.53	2.96–3.87	8.73–9.92
SD	2.89	2.51	1.25	1.65	3.89	5.08
K–Wy	133.55^†^	47.35**	13.99	9.21	5.69	39.49•
K–Wc	77.15^†^	154.24^†^	198.65^†^	228.42^†^	232.49^†^	209.60^†^
*25–74 ALL*						
CI	16.07–17.74	8.93–10.06	4.15–5.11	2.47–3.39	7.45–9.77	23.66–26.81
SD	7.15	4.82	4.13	3.93	9.94	13.48
K–Wy	153.18^†^	24.37	16.06	8.75	5.78	19.04
K–Wc	65.54^†^	193.86	227.13^†^	248.13^†^	237.86^†^	233.49^†^

*K–Wy* Kruskal–Wallis test *χ*^2^ by year, *K–Wc* Kruskal–Wallis test *χ*^2^ by country

•*p* value < 0.1; *p value < 0.05; **p value < 0.01; ^†^*p* value < 0.001

As mentioned above, it is appropriate to take into account the fact that the significant difference between years and between countries was confirmed. In order to transfer the effect of the difference to the regression analysis below, the cluster analysis was carried out. This analysis included total mortality data for the population under 74 years (inclusive).

When applying cluster analysis for individual years, the Ward’s method was considered as the most appropriate method based on the agglomerative coefficient (0.97). The Silhouette method recommended two clusters, thus in terms of the relations between assessment of the economic and health dimension, the years were divided into two groups: (1) 1990–2002, (2) 2003–2016. As regards individual countries, in the Ward’s method the agglomerative coefficient acquired a less satisfactory value (0.86) than in the previous case, but this value was the highest of several validated methods and was also acceptable.

The application of regression models was preceded by testing of assumptions, the F test for individual effects verified the significance of the effect of the variable created by the cluster analysis, and the Hausman Test verified the preference of the fixed effect model or the random effect model. The constancy of variability of residuals was tested using the Breusch–Pagan test, and in the case of significant heteroscedasticity, the Arellano estimator for the fixed effect model and the White 1 estimator for the random effect model were applied.

Table 2 shows the outputs of testing the assumptions about the existence of the effect of HDI on PCMi and the effect of PCMi on GDP in the classification of age groups (children: 0–14 years, adolescents: 15–24 years, adults: 25–74 years) and in the classification of males, females and the population regardless of gender. The *p* value was the most important element in determining the significance of relations, while the relationship was considered significant if the *p* value was less than 0.05. The *R*^2^ was considered as a comparative metric.

**Table 2:** Regression analysis outputs—health and economic indicators in the countries of Central and Eastern Europe (1990–2016)

Regression analysis	MALE						FEMALE						ALL					
	HDI- > PCMi			PCMi- > GDP			HDI- > PCMi			PCMi- > GDP			HDI- > PCMi			PCMi- > GDP		
	R2	alpha	Beta	R2	alpha	Beta	R2	alpha	Beta	R2	alpha	Beta	R2	alpha	Beta	R2	alpha	Beta
*0–14*																		
TRANSPORT	0.10w	16.17^†^	− 14.73^†^	0.03a	–	− 286.88•	0.07w	8.12^†^	− 6.55**	0.02a	–	− 321.19	0.09w	12.01^†^	− 10.44^†^	0.03a	–	− 369.68
FALLS	0.00a	–	0.64	0.01a	–	− 455.09	0.00a	–	0.68	0.00a	–	− 197.76	0.00a	–	0.66	0.00a	–	− 516.73
DROWNING	0.11w	18.77^†^	− 18.39^†^	0.06r	9345.40^†^	− 407.28^†^	0.08w	7.54^†^	− 7.50^†^	0.04w	8687.84^†^	− 693.10^†^	0.12w	13.26^†^	− 13.03^†^	0.07w	9575.53^†^	− 652.32^†^
FIRE	0.02w	3.06*	− 2.70•	0.00r	7760.40^†^	− 320.03	0.04w	3.84^†^	− 3.97**	0.01r	7877.90^†^	− 543.33*	0.03w	3.82**	− 3.29**	0.01r	7889.19^†^	− 469.69•
POISONING	0.04w	3.25^†^	− 3.20**	0.02a	–	− 570.00	0.00a	–	− 0.76	0.01	–	− 414.01	0.00a	–	− 1.54	0.01a	–	− 602.64
SELF_HARM	0.03r	0.53	0.49	0.00w	7641.74^†^	− 217.02	0.03w	0.28	0.03	0.00w	7449.38^†^	− 25.04	0.03r	0.36	0.33	0.00w	7627.46^†^	− 297.74
*15–24*																		
TRANSPORT	0.03w	58.77^†^	− 37.76*	0.00a	7967.96^†^	− 17.65	0.03w	11.98*	− 5.02	0.02w	8604.55^†^	− 142.86*	0.02w	34.53^†^	− 20.04•	0.00w	8162.01^†^	− 37.57
FALLS	0.001a	–	3.53	0.01w	8205.05^†^	− 284.74*	0.03r	0.80	− 0.20	0.00f	–	− 380.12	0.01a	–	2.14	0.01w	8245.17^†^	− 475.86*
DROWNING	0.09w	25.82^†^	− 24.54^†^	0.03w	8889.58^†^	− 206.52^†^	0.06w	3.65^†^	− 3.52**	0.01f	–	− 564.38•	0.08w	14.43^†^	− 13.57^†^	0.03w	8932.94^†^	− 370.01^†^
FIRE	0.02w	1.63•	− 1.05	0.00r	7590.51**	− 178.37	0.02r	1.04*	− 1.05•	0.00r	7577.91**	− 588.82	0.02w	1.35*	− 1.07	0.00r	7647.75**	− 383
POISONING	0.02w	− 8.89	18.96*	0.00r	7308:16**	23.412	0.02w	0.09	1.57	0.00r	7557.07**	− 89.38	0.02w	− 4.77	10.80*	0.00r	7324.67**	32.91
SELF_HARM	0.02w	2.93	22.67*	0.00w	6840.87**	29.54	0.03r	0.90**	− 3.79	0.00r	6999.00**	110.95	0.02w	4.25	10.53•	0.00w	6692.02**	60.76
*25–74*																		
TRANSPORT	0.09w	85.92^†^	− 73.01^†^	0.10w	10,933.49^†^	− 116.55^†^	0.06w	16.84^†^	− 12.65**	0.10w	10,739.94^†^	− 561.44^†^	0.08w	48.48^†^	− 39.85^†^	0.10w	10,934.34^†^	− 194.62^†^
FALLS	0.01w	10.66	7.19	0.01a	–	− 455.09	0.01w	7.95**	− 5.31	0.00f	–	− 197.76	0.01w	8.14	1.99	0.00a	–	− 516.73
DROWNING	0.04w	26.01^†^	− 20.58**	0.03w	9009.65^†^	− 152.76^†^	0.03w	4.05^†^	− 2.85**	0.02w	8861.83^†^	− 763.27^†^	0.04w	13.97^†^	− 10.67**	0.03w	9065.29^†^	− 280.83^†^
FIRE	0.02w	15.98**	− 12.32*	0.02w	8354.20^†^	− 139.90^†^	0.03w	4.86**	− 4.01**	0.02w	8374.30^†^	− 522.24^†^	0.02w	9.81**	− 7.61*	0.02w	8419.93^†^	− 246.12^†^
POISONING	0.01r	4.92	18.20	0.01w	8379.80^†^	− 49.76*	0.02r	0.62	5.01•	0.00w	8158.51^†^	− 160.86•	0.02r	2.07	11.86	0.01w	8352.55^†^	− 81.71*
SELF_HARM	0.01w	28.33	22.99	0.00w	7692.47^†^	− 5.4691	0.01w	13.93•	− 5.75	0.00w	7073.63**	38.59	0.01w	17.31	12.14	0.00w	7375.51^†^	2.46

*f* fixed, *r* random, *a* fixed Arellano, *w* random White 1

•*p* value < 0.1; *p value < 0.05; **p value < 0.01; ^†^*p* value < 0.001

The most significant effects of HDI on fatal transport accidents were found in males in the group of children aged 0–14 years (*α* = 16.17^†^; *β* = − 14.73^†^). Overall, it can be concluded that adolescents showed the least significant effects. In all cases, an inverse effect was confirmed. Based on this, if human development increases, a decrease in fatal transport accidents can be expected, while the greatest effect is expected in the groups of children and adults, in the group of adolescents, the effect is lower. The significant effects of fatal transport accidents on GDP were found in adults (with the most significant effect) and female adolescents (*α* = 8604.55^†^; *β* = − 142.86*), and all cases of fatal transport accidents showed a negative coefficient. Therefore, the economic growth is expected to increase with decreasing fatal transport accidents.

In the vast majority of cases, the relation between fatal falls and economic indicators cannot be considered as significant. The only significant relations were found in the case of the effect of fatal falls on GDP in the group of male adolescents (*α* = 8205.05^†^; *β* = − 284.74*) and in the group of adolescents regardless of gender (*α* = 8245.17^†^; *β* = − 475.86*). In these cases, the inverse relation was confirmed; therefore, the economic growth is expected to increase with decreasing fatal falls in mentioned age groups.

Regarding the relations between economic indicators and drowning, the significant inverse effect was found in almost all cases. The strongest effects were found in children, and the intensity of this effect decreased with increasing age. In terms of gender, males showed the most intensive effects.

Focusing on the relations between economic indicators and fatal injuries due to exposure to smoke, fire and flame, significant relations were found in adults and in several cases in children. The significance was not found in adolescents. The inverse effect was confirmed in all cases; therefore, if the human development increases, deaths due to fire injuries are reduced, and consequently, the economic growth is expected to increase.

Regarding the relations between economic indicators and accidental poisoning, the significant effects were found in only a few cases. In terms of the relation between HDI and poisoning, the significant inverse effect was found in males in the group of children (*α* = 3.25^†^; *β* = − 3.20**), and the positive effect was found in male adolescents (*α* = − 8.89; *β* = 18.96*). Based on this, if the human development increases, a decrease in deaths of male children and an increase in deaths of male adolescents are expected. The inverse effect of poisoning on GDP was found in adult males (*α* = 8379.80^†^; *β* = − 49.76*) and adults regardless of gender (*α* = 8352.55^†^; *β* = − 81.71*). It can be interpreted similarly to previous cases; therefore, the economic growth is expected to increase with declining mortality due to poisoning.

With a focus on the relation of economic indicators and intentional self-harm, only one relation revealed the effect. This was a positive effect of HDI on deaths due to self-harm in the group of male adolescents (*α* = 2.93; *β* = 22.67*); therefore, the frequency of self-harm deaths is expected to increase with increasing human development.

## Discussion

Reducing the burden of fatal injuries is one of the most important challenges for public policies worldwide, with the CEECs being no exception. This general fact is based on economic losses (Harlan et al. 1990; Corso et al. 2006; Geraerds et al. 2019), but also on unjustified preventable deaths. Adolescents with a higher risk of injury and higher injury costs may play a key role in this issue (Harlan et al. 1990; Steinberg 2004, 2007). Based on this, the study focused on evaluating the relations between economic indicators and preventable injury mortality in the CEECs, with a primary focus on adolescents.

In terms of gender, this study revealed that males showed a higher frequency of fatal injuries than females, this fact is in line with findings of many other studies (Yin et al. 2015; Dandona et al. 2020; Ray et al. 2020). Also, the results confirmed that gender differences increase with increasing age (Cunningham et al. 2018). The most pronounced gender differences among adolescents were identified in self-harm (CI-Males: 17.44–20.02; CI-Females: 3.48–3.95) and drowning (CI-Males: 5.11–6.21; CI-Females: 0.70–0.87). Ray et al. (2020) also revealed the predominance of male adolescents in self-harm deaths over their female counterparts, and at the same time, according to Hawton et al. (2012) self-harm is considered a serious health problem, especially in adolescents. Similar findings were demonstrated by Cunningham et al. (2018), who showed that the mortality of male children and adolescents due to drowning is 2.5 times higher compared to female children and adolescents. With a focus on differences between age groups, it can be concluded that the frequency of fatal injuries increases with age, while adults have a negative prevalence, especially in fatal injuries due to fire (CI 2.47–3.39). These results can be compared with the findings of authors such as Corso et al. (2006) and Ray et al. (2020).

The results of this study provide a more detailed view of the examined issue. It is not possible to overlook the inverse effect of human development on fatal transport accidents in all analysed cases and the intensity of this effect is highest in children and adults and incomparably lower in adolescents. In general, the development of society is accompanied by a positive development of human capital, and therefore, this finding is consistent with other studies that confirmed a declining trend of fatal transport accidents during the past decades (Hong et al. 2011; Melchor et al. 2015). This may reflect that people are more educated, have a higher income and the technological progress made it possible to produce vehicles at a higher level of safety. On the other hand, the positive effects of HDI on deaths due to poisoning and intentional self-harm were found in male adolescents, which may be the result of a rapidly changing world and higher demands on the young generation. Several other studies have also confirmed that the economic aspects can affect the injury mortality (Trujillo et al. 2010; Muazzam and Nasrullah 2011).

This study also offers the opposite point of view and examines the effect of fatal injuries on economic growth. As demonstrated in the theoretical background, there are only older international studies that confirmed the economic effects of fatal injuries, but the effect of time was not captured and the sample was different from the CEECs. In this study, the inverse effect of fatal transport accidents on economic growth was evident in adults and female adolescents (*α* = 8604.55^†^; *β* = − 142.86*). The inverse effect of fatal falls on GDP was also found only in groups of adolescents (Males: *α* = 8205.05^†^; *β* = − 284.74*; All: *α* = 8245.17^†^; *β* = − 475.86*). In terms of the relations between economic indicators and drowning, not only adolescents but also the other two age groups showed significant inverse effects, while the intensity of relations decreases with increasing age. On the other hand, adolescents did not show the significant relations between fatal fire injuries and economic indicators, compared to the other age groups. In general, it can be concluded that these findings are in accordance with the results presented by Moniruzzaman and Andersson (2005), who pointed out the fact that the intensity of the effect of fatal injuries on GNP changes irregularly with age. Accordingly, the effect is stronger in children, the adolescents show less intensive effect, subsequently the intensity of this effect increases in adults under 45 years of age and then the effect decreases again. Overall, the individual interpreted findings confirmed the inverse relations between preventable injury mortality and economic growth. These results are consistent with the results of studies that deals with relations between fatal injuries and GNP (Plitponkarnpim et al. 1999; Ahmed and Andersson 2000; Moniruzzaman and Andersson 2005). As indicated in the presented results, the adult population had a strong inverse effect on economic growth, which is consistent with the mentioned studies.

This study provides an interesting insight into the relationship between economic development and preventable injury mortality. In order to increase economic development and to reduce fatal injuries among the population, public policies should focus on this interconnected relationship. Policy makers, who face many economic challenges, need to take into account different age and gender groups as well as the individual causes of injuries. With regard to accidental drowning that showed a significant effect in almost all cases, policy makers should focus more intensively on the younger population (including children and adolescents). In the group of adolescents, public policies should also be active in fall prevention measures. In the population group of adults, fatal injuries due to transport accidents, fire, accidental poisoning are considered to be very significant in economic discussions. Adolescents should not be forgotten when increasing human development, as the increase in deaths from self-harm and poisoning was associated with an increase in HDI. Preventive actions seem to be a key factor that can help adolescents overcome the challenges of this period of life (Orosova et al. 2006). Accordingly, injury prevention and control programs are needed and should be one of the main measures to achieve sustainable development.

We also recommend taking into account the effects of time and countries in similar analyses focusing on the relations of economic and health indicators between different countries and over a period of time. In particular, the “country” identifier is considered significant and failure to take it into account could affect the outputs.

The limitations of the presented research include the fact that that the findings relate to a certain sample of countries that is relatively specific. Therefore, the generalization is useful for similar countries. The time series was included in the analysis, i.e. there were possible risks of hidden effects, but the effects were evaluated to some extent in regression models, and therefore, the outputs can be considered relevant. Future research will focus on verifying the identified assumptions in a sample of other countries and groupings of countries belonging to international organizations.
